# L-type calcium channel blockade worsens glucose tolerance and β-cell function in C57BL6/J mice exposed to intermittent hypoxia

**DOI:** 10.1152/ajpendo.00423.2023

**Published:** 2025-01-06

**Authors:** Stanley M. Chen Cardenas, Tess A. Baker, Larissa A. Shimoda, Ernesto Bernal-Mizrachi, Naresh M. Punjabi

**Affiliations:** 1 Division of Endocrinology, Diabetes, and Metabolism, Johns Hopkins University School of Medicine, Baltimore, Maryland, United States; 2 Division of Endocrinology, Diabetes, and Metabolism, Diabetes Research Institute, Miller School of Medicine, University of Miami, Miami, Florida, United States; 3 Division of Pulmonary, Critical Care, and Sleep Medicine, Johns Hopkins University School of Medicine, Baltimore, Maryland, United States; 4 Division of Pulmonary, Critical Care, and Sleep Medicine, Miller School of Medicine, University of Miami, Miami, Florida, United States

**Keywords:** calcium channel blockade, glucose tolerance, insulin resistance, intermittent hypoxia, sleep apnea

## Abstract

Intermittent hypoxemia (IH), a pathophysiologic consequence of obstructive sleep apnea (OSA), adversely affects insulin sensitivity, insulin secretion, and glucose tolerance. Nifedipine, an L-type calcium channel blocker frequently used for the treatment of hypertension, can also impair insulin sensitivity and secretion. However, the cumulative and interactive repercussions of IH and nifedipine on glucose homeostasis have not been previously investigated. Adult male C57BL6/J mice were exposed to either nifedipine or vehicle concurrently with IH or intermittent air (IA) over 5 days. IH exposure entailed cycling fractional-inspired oxygen levels between 0.21 and 0.055 at a rate of 60 events/h. Nifedipine (20 mg/kg/day) or vehicle was administered via subcutaneous osmotic pumps resulting in four groups of mice: IA-vehicle (control), IA-nifedipine, IH-vehicle, and IH-nifedipine. Compared with IA (control), IH increased fasting glucose (mean Δ: 33.0 mg/dL; *P* < 0.001) and insulin (mean Δ: 0.53 ng/mL; *P* < 0.001) with nifedipine having no independent effect. Furthermore, glucose tolerance was worse with nifedipine alone, and IH further exacerbated the impairment in glucose disposal (*P* = 0.013 for interaction). Nifedipine also decreased glucose-stimulated insulin secretion and the insulinogenic index, with addition of IH attenuating those measures further. There were no discernible alterations in insulin biosynthesis/processing, insulin content, or islet morphology. These findings underscore the detrimental impact of IH on insulin sensitivity and glucose tolerance while highlighting that nifedipine exacerbates these disturbances through impaired β-cell function. Consequently, cautious use of L-type calcium channel blockers is warranted in patients with OSA, particularly in those at risk for type 2 diabetes.

## INTRODUCTION

Obstructive sleep apnea (OSA) is a prevalent disorder that affects 9%–38% of adults in the general population (1). Characterized by upper airway collapse during sleep, OSA has been shown to be an independent risk factor for insulin resistance, glucose intolerance, and type 2 diabetes (2, 3). Experimental evidence from murine models and healthy volunteers demonstrates that exposure to intermittent hypoxia acutely decreases glucose tolerance, insulin sensitivity, and insulin secretion (4–6). Increased oxidative stress, activation of the sympathetic nervous system, and subclinical inflammation are some of the potential mechanisms that could explain the metabolic abnormalities associated with intermittent hypoxemia (IH) (7, 8).

Systemic hypertension is a common comorbidity in patients with OSA (9, 10). Although treatment of OSA with positive airway pressure has been shown to decrease blood pressure, the effects are, at best, modest, with a 2–3 mmHg drop in systolic and diastolic blood pressure (11). In contrast, it is well established that antihypertensive agents, such as dihydropyridine L-type calcium channel blockers (e.g., nifedipine), can have a considerably favorable impact on blood pressure (12). Although highly effective in controlling blood pressure, some antihypertensive agents may also impair glucose metabolism. In fact, calcium channel blockers have been shown to decrease insulin secretion through their effects on L-type calcium channels located on the pancreatic β-cell (13–15). However, the effects of L-type calcium channel blockers on glucose metabolism are a subject of significant controversy, as some studies suggest a beneficial impact whereas others reveal a deleterious one (16–22). Clinical trials in hypertension show that calcium channel blockers may increase the risk of type 2 diabetes (23, 24). Research on the effects of L-type calcium channel blockers on glucose metabolism in murine (25, 26) and in vitro models (27–29) has also been inconclusive. Given that OSA can adversely impact glucose homeostasis (2), the use of L-type calcium channel blockers in these patients could have adverse metabolic sequelae. To examine the effects of an L-type calcium channel blocker on glucose metabolism in OSA, the current study used a murine model to characterize the independent and interactive influence of intermittent hypoxemia and nifedipine on fasting glycemia, glucose tolerance, insulin sensitivity, and β-cell function. Furthermore, to assess whether intermittent hypoxemia and L-type calcium channel blockade adversely alter islet function, the current study also examined the ratio of islet-to-acinar cells and markers of insulin biosynthesis and processing.

## MATERIALS AND METHODS

### Modeling the Effects of Intermittent Hypoxia and Calcium Channel Blockage

**Figure 1.**
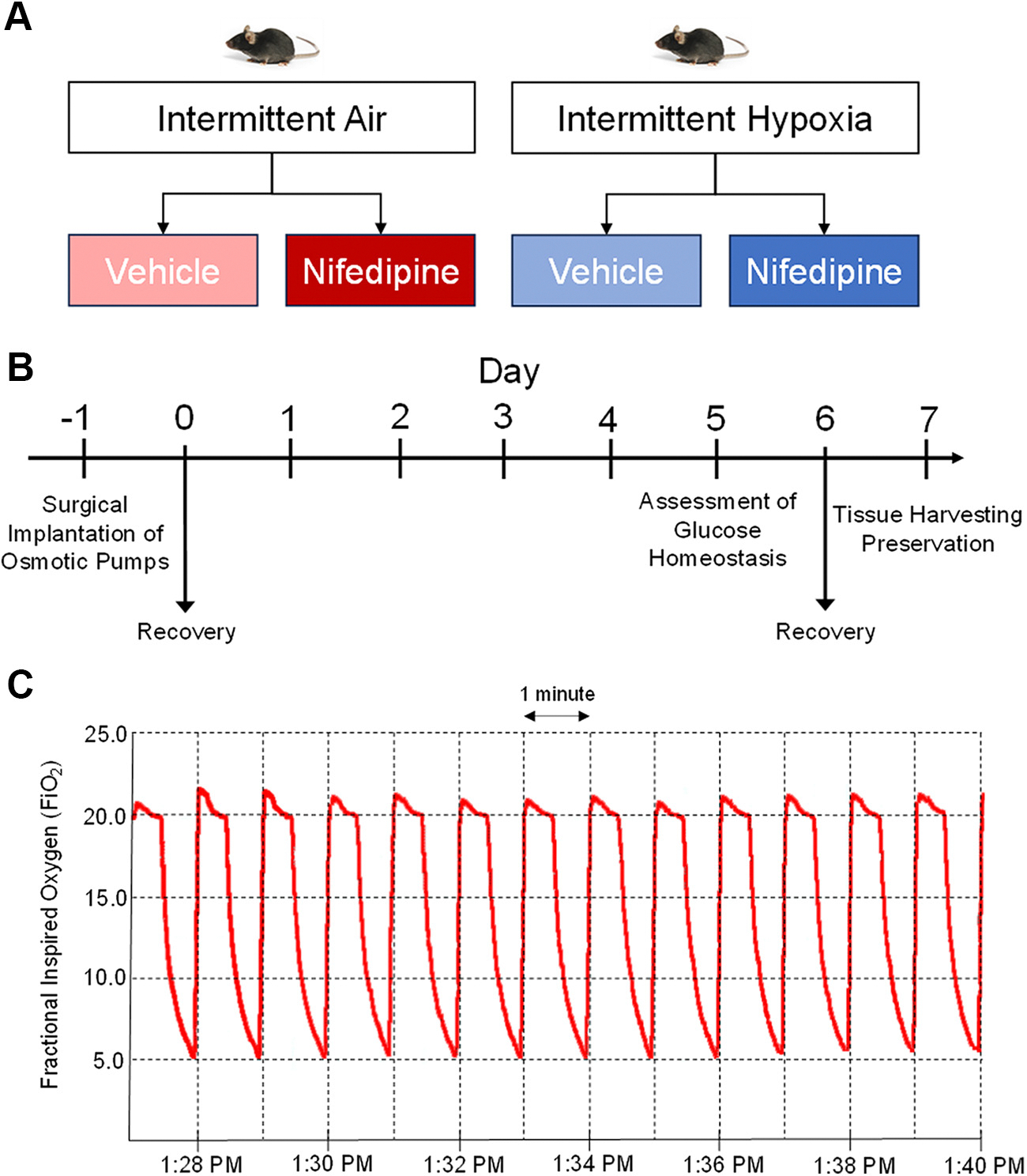
Schematic for the experimental groups (*A*), time course of procedures (*B*), and a illustrative tracing of fractional inspired oxygen from one experiment (*C*).

Adult C57BL6/J (age: 15–22 wk) male mice (Jackson Laboratory, ME) were used and housed in custom-modified cages connected with plastic tubing to a gas control delivery system regulating the flow of nitrogen, oxygen, and compressed air into cages, as previously described (5, 6). Intermittent hypoxemia was induced by programmable solenoid valves and flow regulators, which were used to alter the composition of the gas within the cages. To model intermittent hypoxia (IH) as observed in patients with OSA, the inspired fraction of oxygen (FIO2) was reduced from 0.21 to 0.055 over a period of 30 s and rapidly returned to 0.21 during the following 30 s. For the intermittent air (IA) or the control condition, animals were exposed to alternating periods of air (FI_O_2__ = 0.21), simulating a pattern of airflow at intervals comparable with that of IH. On average, 60 episodes of O_2_ desaturation were induced per hour. All animals were housed at room temperature and subjected to a 12-h light/dark cycle. Exposure to IH or IA was conducted for 12 h during the mouse light cycle (7:00 AM to 7:00 PM). At the end of the exposure period, glucose metabolism was assessed, as described below (Fig. 1). In a subset of experiments, animals were then returned to IA or IH for 24 h before harvesting of tissues. All procedures involving animals were approved by the Institutional Committee on Animal Care and Use and followed NIH principles of laboratory animal care.

Nifedipine or vehicle was administered to the animals via osmotic minipumps (Model 2001, Alzet, Cupertino, CA) implanted subcutaneously in the dorsal region under 1%–2% isoflurane anesthesia mixed with oxygen using a vaporizer (E-Z Systems Corporation Palmer, PA). Pumps were filled with 200 μL of polyethylene glycol 400 (PEG400; Item No. 1008414 Rigaku Reagents, WA) or nifedipine (125 mg/5 mL dissolved in PEG400; Cat. No. 1463508 Sigma-Aldrich). Before implantation, pumps were primed in sterile 0.9% saline at 37°C for 12–18 h. The osmotic pumps delivered contents at a constant flow rate of 1 μL/h resulting in a dose of 20 mg/kg/day of nifedipine in each animal (30). This dose has been previously used in murine studies investigating vascular and metabolic effects, including research on abdominal aortic aneurysm (AAA), where it significantly reduced blood pressure and prevented AAA formation while preserving endothelial function. The dose administered in the current study was chosen to achieve plasma concentrations comparable with those seen in clinical practice, where typical doses range from 30 to 120 mg/day.

### Intraperitoneal Glucose Tolerance Test and Insulin Tolerance Test

After five days of exposure to IH or IA, an intraperitoneal glucose tolerance test (ipGTT) was performed following a 5-h fast under the following four conditions: IA-vehicle, IA-nifedipine, IH-vehicle, and IH-nifedipine (*n* = 10/group). Following a 15% glucose injection [1 g/kg dissolved in phosphate buffered saline (PBS)], glucose levels were measured in tail-snip blood samples at 0, 10, 20, 30, 60, 90, and 120 min using a glucometer (Freestyle InsuLinx, Abbott, IL). An insulin tolerance test (ITT) (*n* = 5/group) was similarly performed after a 2-h fast by measuring blood glucose levels at 0, 10, 20, 30, 40, 50, 60, 90, and 120 min after injecting 0.5 IU/kg of insulin (Humulin R, 100 U/mL, Eli Lilly, IN). The ipGTT (*n* = 40) and ITT (*n* = 20) measurements were performed in a distinct set of freely moving animals with continued IH or IA exposure.

### Glucose-Stimulated Insulin Secretion, Insulin Sensitivity, and β-Cell Function

Glucose-stimulated insulin secretion (GSIS) (*n* = 10/group) was assessed by measuring insulin levels using the Mouse Ultrasensitive ELISA kit (Cat. No. 90080, Crystal Chem, IL) on plasma from tail-snip blood samples from the four groups. Samples were obtained after 5 h of fasting, 15 and 30 min after a 1 g/kg glucose injection. Blood was collected in heparinized capillary tubes (Microvette CB300LH, Sarstedt, Germany), centrifuged at 5,000 rpm for 10 min to separate plasma from whole blood. The plasma was transferred to a clean tube and samples were frozen immediately and kept at −80°C until analysis. Homeostasis model assessment of insulin sensitivity (HOMA%S) and homeostasis model assessment model of β cell function (HOMA%B) values (31) were calculated with the HOMA2 calculator provided by Diabetes Trials Unit, University of Oxford: HOMA Calculator (http://www.dtu.ox.ac.uk/homacalculator/index.php) (32).

### Whole Pancreas Insulin Extraction and Measurement of Insulin and Proinsulin Content

Dissected, snap-frozen pancreata were removed from −80°C and blotted on filter paper before weighing. Thereafter, they were placed in 3 mL of acid ethanol. Exposure time to room temperature was avoided by placing the samples in ice-cold acid ethanol. Acid ethanol was prepared by adding the following: 37.5 mL of 100% ethanol, 11.75 mL of deionized H_2_O, and 700 μL of 12 N HCl. Tissues were then sonicated (rinsing the sonicator in-between tissues) in acid ethanol for 10-s intervals, three times on ice, and left for 48 h rocking in 4°C in a cold room. After 48 h, digested pancreata were centrifuged at 2,400 rpm for 30 min, and supernatant (~3 mL for all tubes) was collected and kept at −80°C until an ELISA for insulin was conducted. Insulin content was measured using the supernatant at 1:1,000 using an ELISA (Alpco 80-INSMSU, Salem, NH). The insulin values were divided by the pancreata mass and final supernatant volume to calculate the final pancreatic insulin content. In addition, proinsulin content was measured and the supernatant was stabilized with 1:1 1 M Tris at a pH of 7.4. Stabilized supernatant was additionally diluted at 1:300 with 1× PBS to fit within the standard curve. Proinsulin ELISA (Mercodia 10-1118-01 Winston-Salem, NC) was used to measure proinsulin content.

### Immunofluorescence Staining and Islet Morphometry

Pancreata from mice exposed to the four different conditions were removed and placed in a 1.5-mL vial, and snap-frozen in liquid nitrogen. Upon the time of experimentation, pancreata were then thawed in 4% paraformaldehyde overnight, and then transferred to 30% sucrose for final overnight fixation. After final fixation, pancreata were embedded in optimal cutting temperature compound (Tissue-Plus O.C.T. Compound 23-730-571 Waltham, MA) and solidified at −80°C. OCT blocks were sectioned at a 40-μm thickness via cryostat slicing. The routine protocol for cryostat sectioning can be reviewed in Ref. 33. Sections were covered and incubated for 48 h at 20°C (room temperature) with primary antibodies: guinea pig anti-insulin (Dako IR00261-2 Santa Clara, CA) and mouse anti-glucagon (Sigma-Aldrich G2654-100UL St. Louis, MO). Sections were then incubated with fluorophore-conjugated secondary antibodies (Thermo Fisher Scientific A-11073 and A-11011 Waltham, MA) and mounting media containing DAPI (Vector Laboratories H-1800-2 Burlingame, CA) was applied, after which coverslips were added. Finally, the islet/acinar ratio was calculated by measuring the number of islets per stained section and dividing by acinar measurements using Adobe Photoshop software (v.22.4.2).

### Statistical Analysis

Data are reported as medians with associated interquartile ranges along with the mean and standard error of the mean. Normality was tested using the Shapiro–Wilk test. Homeostasis model assessment of insulin resistance (HOMA-IR), HOMA2-%S, and HOMA2-%B were calculated and log-transformed given their skewed distribution. Differences between groups were then assessed for fasting glucose, insulin, HOMA-IR, HOMA2-%S, HOMA2-%B, and the GTT- and ITT-derived parameters using analysis of variance. For data derived from the ipGTT and ITT, the area under the glucose and insulin curves (AUC) was also determined for each of the exposure conditions using the trapezoidal rule. To assess the independent and interactive effects of IH and nifedipine, generalized linear models with robust standard errors were used with IH and nifedipine as the main effects, along with an interaction term between the two. All analyses were performed using Stata 17.0 and SAS 9.4 statistical software packages. A *P* value <0.05 was considered statistically significant.

## RESULTS

### Effects of Intermittent Hypoxia and Nifedipine on Fasting Glucose and Insulin

**Figure 2.**
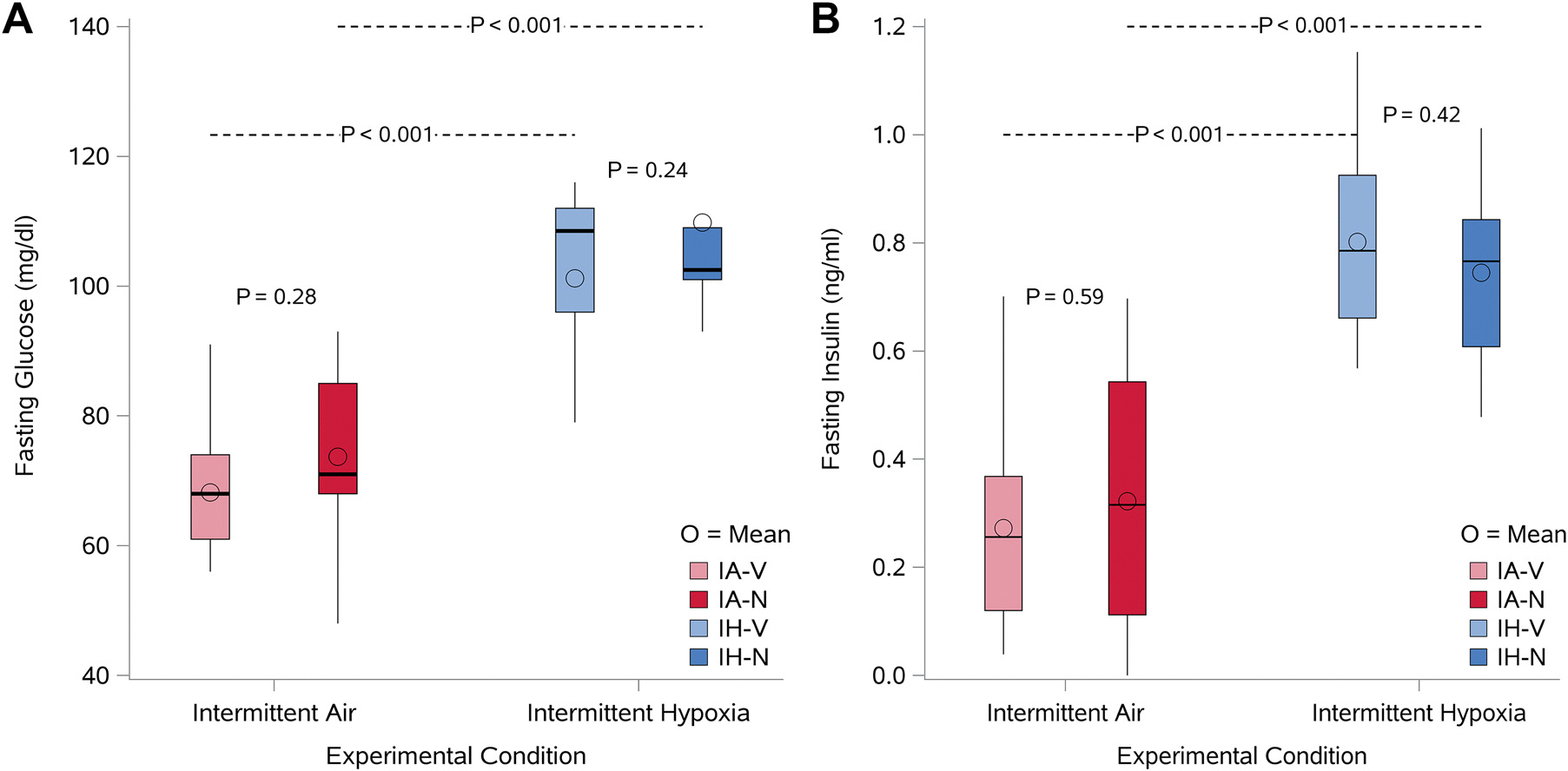
Box plots of fasting glucose (*A*) and fasting insulin (*B*) levels in the four groups of mice (*n* = 10/group). The experimental groups comprised animals exposed to either intermittent air (IA) or intermittent hypoxia (IH) in combination with vehicle (V) or nifedipine (N). Mice were exposed to IA or IH for 12 h daily over five consecutive days, with vehicle or nifedipine (20 mg/kg/day) administered via subcutaneous osmotic pumps. Fasting blood samples were collected after 5 h of fasting, following the exposure period. The four groups included: IA-vehicle (IA-V), IA-nifedipine (IA-N), IH-vehicle (IH-V), and IH-nifedipine (IH-N). The box plots depict fasting glucose (mg/dL) and fasting insulin (ng/mL) levels for each experimental group. Sample size is *n* = 10/group.

Table 1 shows the weights in mice at baseline and at the end of the exposure in the four groups. Baseline and final weights were comparable across the four groups, with minor differences in weight over the exposure period (Supplemental Table S1), indicating that pair-feeding the mice across different conditions was successful in matching the weight loss observed with IH. Fasting glucose and insulin levels were higher with IH irrespective of whether the animals were treated with vehicle or nifedipine (Fig. 2). IH increased fasting glucose by 33.0 mg/dL (SE: 5.8) and 36.1 mg/dL (SE: 6.7) with vehicle and nifedipine, respectively (Supplemental Table S2). Similarly, fasting insulin levels increased by 0.53 ng/mL (SE: 0.08) and 0.42 ng/mL (SE: 0.09) with IH combined with vehicle and nifedipine, respectively (Supplemental Table S2). Thus, no statistically significant interactions were observed between experimental condition (IH or IA) and drug assignment (vehicle or nifedipine) for glucose (*P* = 0.73) or insulin (*P* = 0.36) values. Given the increase in fasting glucose and insulin levels with IH, HOMA-IR was higher. A similar effect was also observed with nifedipine alone (Table 2); however, an interaction was not noted between IH and nifedipine (*P* value 0.99). To assess insulin sensitivity and β-cell function, HOMA2-%S and HOMA2-%B were determined, respectively. HOMA2-%S was lower with IH, but was not affected by nifedipine (Table 2). Combining nifedipine with IH had no additional effects on HOMA2-%S beyond that observed with IH alone. Finally, HOMA2-%B was not significantly different with either IH or nifedipine alone compared with the control condition.

### Effects of Intermittent Hypoxia and Nifedipine on Glucose Tolerance and Insulin Sensitivity

**Figure 3.**
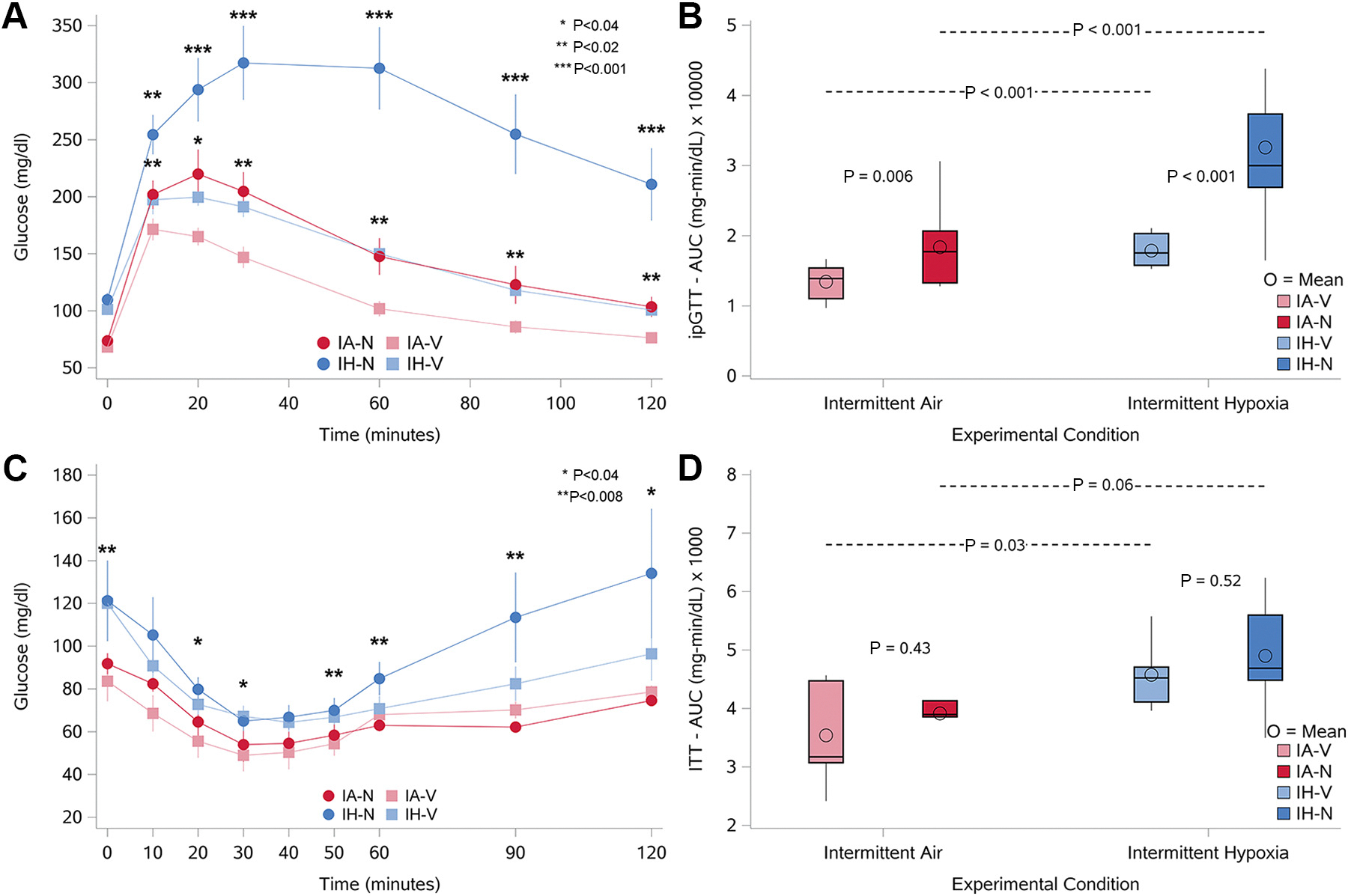
Glucose profiles and area under the curve (AUC) during intraperitoneal glucose tolerance tests (ipGTT) and insulin tolerance tests (ITT) in four groups of animals exposed to either intermittent air (IA; control) or intermittent hypoxia (IH) and treated with either vehicle (V) or nifedipine (N). *A*: glucose profiles during the ipGTT, where mice were fasted for 5 h before receiving an intraperitoneal glucose injection (1 g/kg). Data points represent mean glucose values at each time point, and error bars represent the standard error of the mean (SEM). *n* = 10/group. Statistical significance for comparisons between groups is indicated by *P < 0.04, **P < 0.02, and ***P < 0.001. *B*: box plots of the AUC for the ipGTT, summarizing the total glucose response over time for each group. Significant differences between groups are indicated, with *P* values provided for each comparison. *C*: glucose profiles during the ITT, where animals were fasted for 2 h before receiving an intraperitoneal insulin injection (0.5 IU/kg). Data points represent mean glucose values at each time point, and error bars represent the SE. *n* = 5 per group. Statistical significance for comparisons between groups is indicated by *P < 0.04 and **P < 0.008. *D*: box plots of the AUC for the ITT (mg·min/dL × 1,000), summarizing the total glucose response over time for each group. *P* values for comparisons between groups are indicated. See Tables 3 and 4 for detailed *P* values related to different group comparisons in *A* and *C*, respectively. *n* = 10/group for ipGTT (*A* and *B*), and *n* = 5/group for ITT (*C* and *D*).

Glucose tolerance, as assessed with the ipGTT, was lower in animals exposed to IH, with higher glucose levels at each time starting 10 min after the glucose load (Fig. 3A; dark blue squares and Supplemental Table S3A). A similar degree of impairment in glucose tolerance was also observed in animals exposed only to nifedipine alone (Fig. 3A; maroon circles). When IH was combined with nifedipine (dark blue circles), glucose tolerance was further impaired when compared with either condition alone. Animals exposed to IH and nifedipine exhibited significantly higher glucose levels at all time points after the glucose load when compared with the other groups (Table 3). In addition, glucose disposal was slower when IH was combined with nifedipine than with either condition alone (*P* = 0.02 for interaction between experimental condition and drug), with blood glucose levels remaining significantly higher even 120 min after the glucose load (Table 3) suggesting an impairment in insulin secretion. The AUC from the ipGTT (Fig. 3B and Supplemental Table S3), which quantifies the area under the glucose curve, was increased by nifedipine and IH. Moreover, the AUC from the ipGTT was significantly higher in animals exposed to the combination of IH and nifedipine than in any of the other groups (*P* = 0.013 for interaction). Data from the ITT showed that glucose levels with IH alone were significantly higher than the control condition (IA alone) at fasting and at 20 min and 30 min (Fig. 3C and Table 4). In contrast, animals exposed to the nifedipine alone showed no differences in glucose value during the ITT compared with the control group (IA with vehicle). Significantly higher glucose levels were also observed at time points 50, 60, 90, and 120 min when IH was combined with nifedipine compared with the nifedipine alone condition (Table 4). However, glucose values with IH and nifedipine at time points 50, 60, 90, and 120 min, when compared with IA and nifedipine, were different when compared with IH alone. Consistent with these findings, the AUC from the ITT with IH alone and IH with nifedipine was higher than the control conditions (Fig. 3D and Supplemental Table S3).

### Effects of Intermittent Hypoxia and Nifedipine on Glucose-Stimulated Insulin Secretion

**Figure 4.**
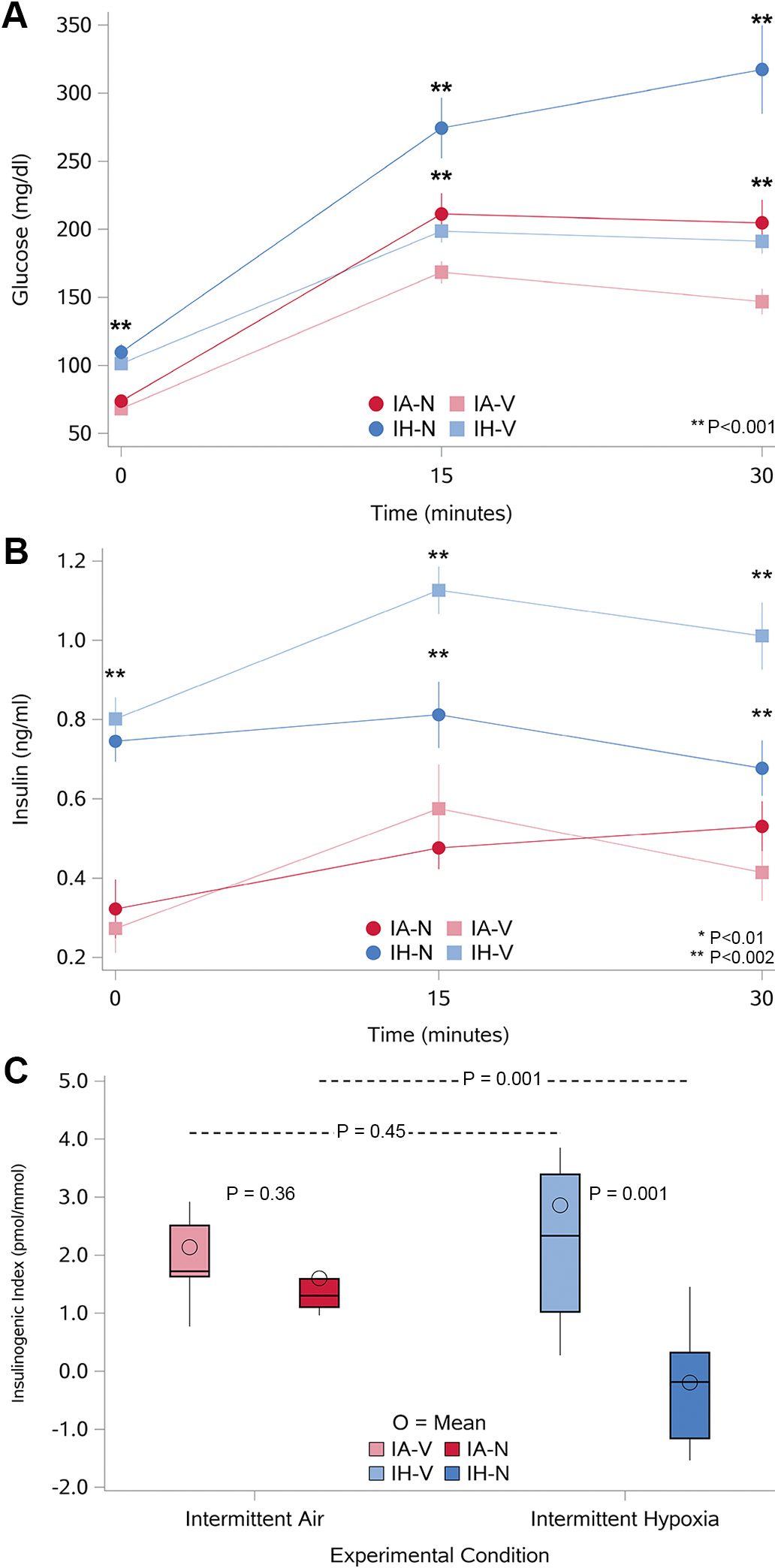
Glucose-stimulated insulin secretion (GSIS) assessed through glucose levels (*A*), insulin levels (*B*), and insulinogenic index (*C*) in the four experimental groups. Mice were fasted for 5 h before receiving an intraperitoneal glucose injection (1 g/kg) to stimulate insulin secretion. Blood samples were collected at 0-, 15-, and 30-min postinjection to assess glucose and insulin levels in each group. *n* = 10/group. *A*: glucose levels (mg/dL) over time during GSIS for each experimental group: intermittent air (IA)-vehicle (IA-V), IA-nifedipine (IA-N), intermittent hypoxia (IH)-vehicle (IH-V), and IH-nifedipine (IH-N). Data points represent mean glucose values, with error bars representing the standard error of the mean (SEM). Significant differences between groups at each time point are indicated by *P < 0.01 and **P < 0.002. *B*: insulin levels (ng/mL) over time during GSIS for each experimental group. Data points represent mean insulin levels, with error bars representing the SE. Insulin levels were significantly elevated in the IH groups compared with the IA groups at all time points (0, 15, and 30 min), whereas nifedipine impaired insulin secretion in the IH groups compared with the IA groups (see Supplemental Table S4 for statistical details). *C*: box plots of the insulinogenic index, calculated as Δ[*I*_30_ − *I*_0_]/Δ[*G*_30_ − *G*_0_], representing insulin secretion relative to the glucose increase over the first 30 min. The insulinogenic index was significantly lower in the IH-nifedipine group compared with the other groups, suggesting a blunted insulin response to glucose stimulation in this group. *P* values for comparisons between groups are indicated. For *P* values related to group comparisons for *A* and *B*, see Supplemental Table S4. *n* = 10/group for all panels.

In vivo GSIS was used to characterize the effects of IH and nifedipine on insulin secretion and β-cell function. As shown in Fig. 4A, glucose values during the GSIS were significantly higher when IH was combined with nifedipine (dark blue circles) compared with the other three groups. The temporal profiles of insulin levels during the GSIS are shown in Fig. 4B. With IA, insulin levels increased at 15 min and returned to near-baseline values by 30 min. The temporal profile of insulin with exposure to nifedipine was comparable with that observed with IA. With IH alone or in combination with nifedipine, insulin levels at baseline were higher compared with the IA or nifedipine-alone conditions. In addition, insulin levels did not return to baseline values with IH alone. Finally, when IH was combined with nifedipine, the expected increase in insulin levels at 15 min was absent and remained unchanged even at the 30-min time point despite higher glucose levels, indicating an impairment in glucose-stimulated insulin release. To further assess the insulin secretory response to glucose, the insulinogenic index (IGI) was determined as the ratio of insulin concentration at 30 min minus fasting insulin to the difference of glucose at the same time (Δ[*I*_30_ − *I*_0_]/Δ[*G*_30_ − *G*_0_]). IGI values from the four groups confirmed the lack of glucose-stimulated insulin secretion exclusively in mice exposed to the combination IH with nifedipine (Fig. 4C).

### Effects of Intermittent Hypoxia and Nifedipine on β-Cell Insulin Content and Islet Morphometry

**Figure 5.**
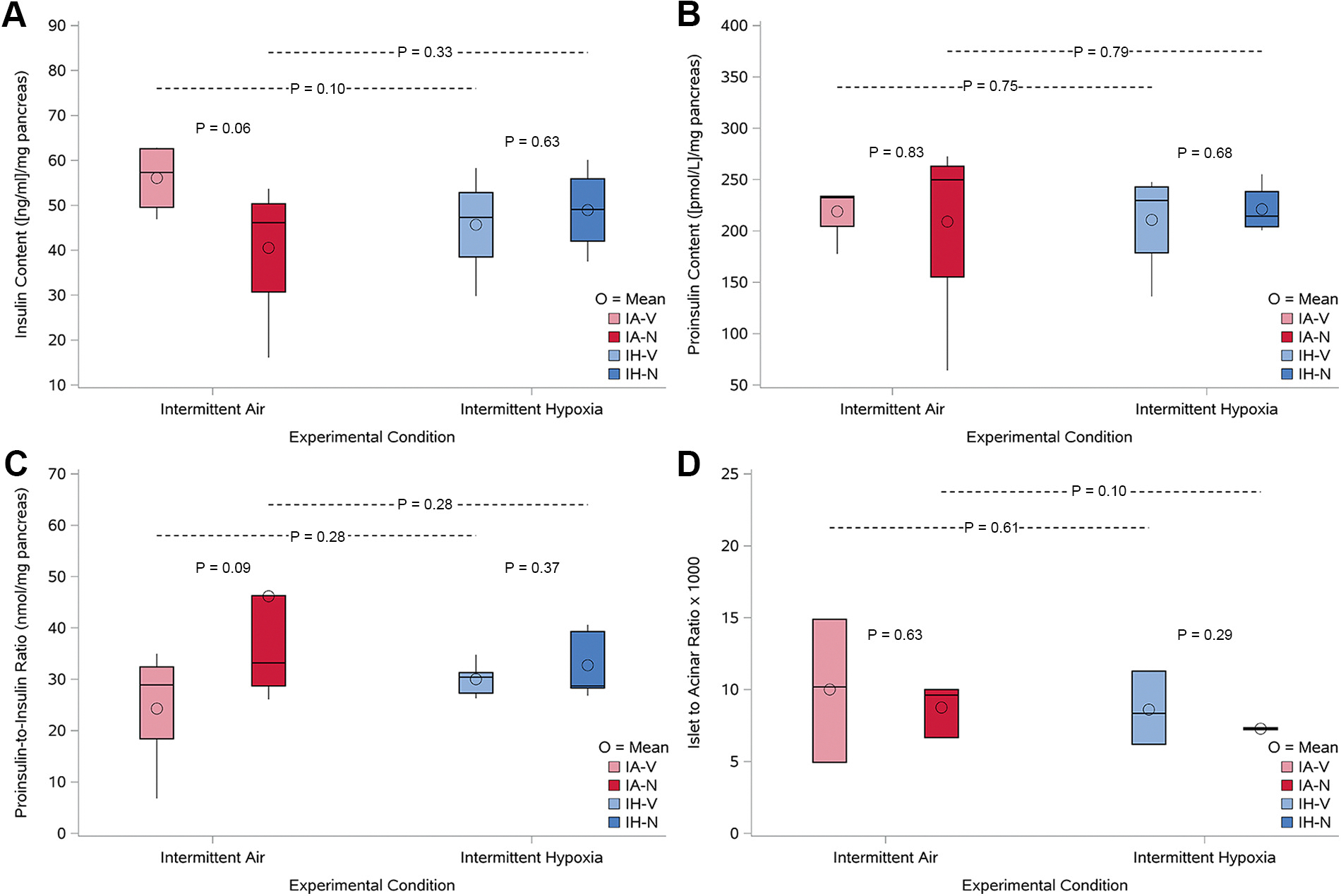
Box plots depicting β-cell insulin content, proinsulin content, proinsulin-to-insulin ratio, and islet-to-acinar ratio in animals exposed to intermittent air (IA) or intermittent hypoxia (IH) and treated with either vehicle (V) or nifedipine (N). Mice were exposed to either IA or IH for 5 consecutive days, with vehicle or nifedipine (20 mg/kg/day) administered via subcutaneous osmotic pumps. Pancreatic tissues were harvested after the exposure period, and insulin and proinsulin contents were measured from pancreatic extracts, while islet morphology was assessed via histology. *A*: insulin content (ng/mg pancreas) in β-cells, assessed from pancreatic tissue in the four experimental groups: IA-vehicle (IA-V), IA-nifedipine (IA-N), IH-vehicle (IH-V), and IH-nifedipine (IH-N). *n* = 5/group. *B*: proinsulin content (pmol/L/mg pancreas) in β-cells, measured from pancreatic tissue extracts. *n* = 5/group. *C*: proinsulin-to-insulin ratio (nmol/mg pancreas) in the four groups, calculated to assess insulin processing efficiency in the pancreatic islets. *n* = 5/group. *D*: islet-to-acinar ratio (×1,000), measured to assess the relative size and structure of the pancreatic islets compared with the surrounding acinar tissue. *n* = 3/group. *P* values for comparisons between groups are indicated in each panel.

Insulin biosynthesis and insulin processing were then examined in islets isolated from all four animal groups. Insulin content, proinsulin content, and the proinsulin/insulin ratio, islet-to-acinar ratio, were similar, suggesting that insulin processing and biosynthesis were not altered by intermittent hypoxia and nifedipine (Fig. 5, A–C). Consistent with the insulin content studies, the insulin/acinar ratio was not different across the four groups (Fig. 5D).

## DISCUSSION

The results of this study demonstrate that IH and nifedipine, an L-type calcium channel blocker, have independent and interactive effects on glucose homeostasis. First, during an ipGTT, IH alone increases fasting glucose and insulin levels. In contrast, nifedipine has no such effect and does not augment the increase in fasting glucose or insulin levels observed with IH. Second, glucose tolerance during the IPGTT was impaired with IH and nifedipine exerting independent effects, and this impairment was further exacerbated by the combination of the two exposures. Third, insulin sensitivity, as assessed by HOMA2-IR or the insulin tolerance test, significantly decreased with IH, with no additional effects of nifedipine. Fourth, IH heightened the insulin response to a glucose load (i.e., GSIS). Combining IH with nifedipine resulted in a distinct phenomenon, where the insulin response to the glucose load was effectively blunted. Finally, IH and nifedipine did not impact β-cell proinsulin or insulin content, nor did they affect overall islet size and morphology. Collectively, these findings suggest that IH detrimentally affects glucose disposal by impairing insulin sensitivity and decreasing glucose tolerance, and nifedipine exacerbates these effects by impairing glucose-stimulated insulin secretion.

Calcium channel blockers are a heterogeneous group of antihypertensive agents with three distinctive subclasses (i.e., phenylalkylamines, benzothiazepines, and dihydropyridines). Of these, the dihydropyridines are the most potent vasodilators and used commonly for the management of hypertension. Evidence on the effects of calcium channel blockers on glucose homeostasis is, at best, mixed, with some studies showing no effect (32, 34–39), an unfavorable effect (40, 41), or a favorable effect (16–22, 42–48). This inconsistency is likely due to several factors, including the type of calcium channel blocker used, the inclusion of combination therapy with other agents [e.g., β-blocker, angiotensin-converting enzyme (ACE), thiazide diuretic] and the target sample (i.e., patients with and without type 2 diabetes). Meta-analyses of hypertension trials indicate that angiotensin receptor blockers (ARBs) and ACE inhibitors have the lowest risk for type 2 diabetes compared with calcium channel blockers (23, 49). Data from the ALLHAT trial show that, compared with an ACE inhibitor, the incident rate ratio for type 2 diabetes for calcium channel blockers was 34% higher (50). A similar elevated risk was observed with calcium channel blockers use in the VALUE trial (51), which revealed that, compared with an ARB, the incidence of type 2 diabetes was 23% higher. These results suggest that there are metabolic disadvantages with calcium channel blockers relative to an ACE-inhibitor or an ARB. Considering the results of this study, which show that IH-related impairments in insulin sensitivity and glucose intolerance can be exacerbated by nifedipine, additional research is needed investigating the metabolic effects of these agents in patients with obstructive sleep apnea and other comorbidities such as hypertension.

The mechanisms through which calcium channel blockers alter glucose homeostasis are not fully defined. Six classes of calcium channels (L, T, N, P, Q, and R) have been identified. Specific calcium channels are located in certain tissues, whereas others are expressed more widely. For example, L-type calcium channels are present in pancreatic β-cells and in mice, have been shown to regulate the first phase of the insulin secretion, given that L-type calcium channels are involved in exocytosis of insulin granules (13–15). In fact, β-cell-specific knockout of the L-type channels (CaV1.2 subtype) can impair insulin secretion and glucose tolerance (14). Other L-type calcium channels (CaV1.3 subtype) have been linked to proliferation and maintenance of β-cell mass (52). Although there are species-specific differences in the L-type channels in β-cells (14, 53), the significance of calcium channels in insulin secretion has been demonstrated. In the clinical setting, patients with calcium channel blocker overdose often demonstrate severe hypoinsulinemia and hyperglycemia that require high doses of insulin to reverse the metabolic effects (54). The current study suggests that the defects in nifedipine-related impairments in glucose-stimulated insulin secretion appear to be functional, given that no differences were noted in insulin content or islet morphology. Furthermore, given that nifedipine had no impact on fasting glucose and insulin levels, but did impair glucose disposal, implies that it may hinder insulin secretion specifically in a postmeal state rather than during fasting.

A plausible mechanism for the effects observed in this study involves the dysregulation of calcium signaling pathways. As a calcium channel blocker, nifedipine reduces intracellular calcium levels, which are essential for the proper functioning of pancreatic β-cells (55). Calcium influx is a critical trigger for insulin granule exocytosis (56). Therefore, impairing this pathway likely diminishes insulin secretion in response to glucose stimulation, contributing to hyperglycemia. Furthermore, chronic exposure to intermittent hypoxia can lead to oxidative stress and inflammation, which activate pathways such as NF-κB and increase the production of proinflammatory cytokines (57). These inflammatory mediators can impair insulin signaling by promoting serine phosphorylation of insulin receptor substrates, reducing the efficacy of insulin in stimulating glucose uptake in peripheral tissues. In addition, there may be a critical interplay between calcium signaling and insulin signaling pathways. Disruption of calcium homeostasis can influence the activity of enzymes such as phosphoinositide 3-kinase (PI3K) and protein kinase B (AKT), which are vital for glucose transport and metabolism in insulin-sensitive tissues such as muscle and adipose tissue (58). Collectively, these molecular pathways provide a multifaceted framework for understanding how disruptions in calcium signaling and the inflammatory response can exacerbate impairments in glucose homeostasis. Further exploration of these mechanisms is warranted to fully elucidate the complex interactions that contribute to the metabolic disturbances observed in our study.

The underlying mechanisms through which IH affects glucose disposal are largely unknown. Intermittent hypoxemia can activate the sympathetic nervous system, impair endothelial function, increase oxidative stress, and heighten systemic inflammation, all of which can act in concert to alter glucose disposal (59–61). Although the relative contribution of each is unknown, there is little doubt that sympathetic activation (62) and oxidative stress have a central role (63, 64). IH also leads to attenuation of glucose-induced insulin secretion from pancreatic β-cells (65, 66) and to enhancement of insulin resistance in hepatocytes, adipocytes, and myocytes (67). Increased oxidate stress, particularly in the islets, can also lead to β-cell dysfunction (64) leading to basal hyperinsulinemia with additional impairments in glucose-stimulated insulin secretion that are potentiated by nifedipine.

The findings of this study regarding the effects of nifedipine on insulin secretion align with prior research, which indicates that calcium channel blockers like nifedipine can modulate insulin secretion in a dose-dependent manner. Studies in rat islet β cells have demonstrated that higher concentrations of nifedipine significantly inhibit calcium influx and insulin secretion under high-glucose conditions, a phenomenon likely driven by reduced calcium availability in islet cells (68). Similarly, research involving ob/ob mice has shown that nifedipine more potently inhibits glucose-induced insulin release compared with other calcium channel blockers, such as verapamil (69). The variation in these inhibitory effects underscores the importance of physiological context and dosage in determining the impact on insulin secretion. Differences across studies may be attributed to factors such as experimental models, the underlying metabolic state, and the methodologies used to assess insulin release. These complexities emphasize the need for further research to fully elucidate the mechanisms underlying these effects.

There are several strengths and limitations of the current study that warrant discussion. One strength is that an established murine model was used in this study to test a novel hypothesis regarding the independent and interactive metabolic effects of IH and nifedipine. Second, weight matching across all the IH and IA groups minimized confounding due to changes in weight common with IH. However, weight matching the control (IA) and IH groups could lead to lower glucose values in the control animals and thereby increase the difference between the IH and IA groups. Third, the metabolic phenotyping used provided insights into alterations induced in glucose homeostasis not only in the basal state, but also after a glucose challenge. Finally, assessing proinsulin/insulin content along with islet size and morphology excludes the possibility that IH and nifedipine, independently or in combination, lead to defects in proinsulin processing, or insulin storage, or in β-cell mass. However, to attain a comprehensive understanding of the interactive impact of calcium channel blockers and IH on glucose metabolism, it is imperative that future studies examine other crucial organ systems integral to glucose homeostasis, including the liver, skeletal muscle, and adipose tissue. Another key limitation of this study is the exclusive use of male mice, which limits its generalizability. The focus on male mice was driven by the need to control for the variability introduced by hormonal fluctuations associated with the estrous cycle, which can affect glucose metabolism and insulin sensitivity (70). This decision allowed for a more controlled experimental model and clearer interpretation of the effects of intermittent hypoxia and nifedipine on glucose homeostasis. However, sex hormones are known to influence metabolic responses and may interact with the pathways affected by intermittent hypoxia and calcium channel blockers (33). Therefore, the findings herein may not fully reflect the potential sex differences in these effects. Future studies are warranted to explore these dynamics in female mice to better understand how sex differences modulate the interaction between intermittent hypoxemia, glucose metabolism, and pharmacological interventions such as nifedipine. Additional weaknesses of the study include the lack of assessment of other calcium channel blockers, the limited duration of exposure, and the absence of mechanistic data on possible alterations induced in β-cell membrane transporters (e.g., voltage-gated calcium channels) or insulin signaling pathways.

The aforementioned weaknesses notwithstanding the results of this study show that the use of nifedipine against a background of intermittent hypoxemia leads to insulin resistance, impaired glucose-stimulated insulin secretion, and glucose intolerance. As these impairments persist, the propensity for developing type 2 diabetes escalates, leading us to ponder the potential ramifications of using calcium channel blockers in the management of hypertensive patients with OSA. Additional research is clearly needed to determine whether, in patients with OSA, calcium channel blockers alter glucose homeostasis and whether treatment of OSA mitigates these effects.

## Supplementary Material

Supplemental Material

Supplemental Tables S1–S5: https://doi.org/10.6084/m9.figshare.28098200.

## Figures and Tables

**Table 1.** Weight under conditions of intermittent air (IA) and intermittent hypoxia (IH) with and without a calcium channel blocker

	Vehicle (V)			Nifedipine (N)				
Weight,* g	IA	IH	*P* Value^†^	IA	IH	*P* Value^†^	Δ_V_ – Δ_N_	*P* Value‡

Baseline	31.73 (0.67)	32.62 (0.86)	0.42	32.48 (0.90)	33.00 (0.89)	0.68	−0.37 (1.67)	0.83
End of exposure	30.18 (0.51)	29.90 (0.72)	0.75	30.49 (0.57)	30.17 (0.74)	0.73	−0.04 (1.28)	0.98
Difference**	−1.55 (0.28)	−2.72 (0.18)	0.001	−1.99 (0.49)	−2.83 (0.27)	0.14	−0.33 (0.65)	0.62

IA, intermittent air; IH, intermittent hypoxia.

* Values represent means and SE

** Difference between the baseline and end-of-exposure weight

† P values derived from generalized linear model with robust variance estimation comparing IH to IA

‡ P value derived from the interaction between experimental condition and drug from a generalized linear model to estimate the difference of differences in weight change between vehicle and nifedipine (Δ_V_ – Δ_N_).

**Table 2.** Fasting measures of insulin sensitivity and insulin secretion under conditions of intermittent air and intermittent hypoxia with and without nifedipine

	Vehicle (V)			Nifedipine (N)				
Variable*	IA	IH	*P* Value^†^	IA	IH	*P* Value^†^	Δ_V_ – Δ_N_	*P* Value‡

Log (HOMA-IR)	1.23 (0.25)	2.53 (0.06)	0.001	1.18 (0.42)	2.49 (0.06)	0.001	−0.01 (0.50)	0.99
Log (hOMA2-%S)	3.38 (0.25)	2.08 (0.06)	0.001	3.42 (0.42)	2.12 (0.06)	0.001	0.01 (0.49)	0.99
Log (hOMA2-%B)	6.04 (0.16)	6.21 (0.10)	0.48	5.87 (0.23)	6.01 (0.10)	0.53	0.02 (0.32)	0.96

HOMA-IR, homeostasis model assessment of insulin resistance; HOMA2-%B, homeostasis model assessment model of β cell function; HOMA2-%S, homeostasis model assessment of insulin sensitivity; IA, intermittent air; IH, intermittent hypoxia.

* Values represent means and SE

† Pvalues derived from analysis of variance comparing intermittent hypoxia to intermittent air

‡ *P* value derived from the interaction between experimental condition and drug from a generalized linear model to estimate the difference of differences between IH and IA with and without nifedipine (Δ_V_ – Δ_N_).

**Table 3.** Effects of intermittent hypoxia and nifedipine on glucose tolerance

Time (min)	Group A vs. Group B		Effect Assessed^†^	Background	Mean Δ (SE)‡	*P* Value*

*T* = 0	IA-N	IA-V	N	IA	5.5 (5.1)	0.28
	IH-V	IA-V	IH	V	33.0 (5.8)	<0.001
	IH-N	IA-N	IH	N	36.1 (6.7)	<0.001
	IH-N	IH-V	N	IH	8.6 (7.3)	0.24
*T* = 10	IA-N	IA-V	N	IA	30.4 (15.2)	0.05
	IH-V	IA-V	IH	V	26.0 (15.3)	0.09
	IH-N	IA-N	IH	N	52.7 (20.5)	0.01
	IH-N	IH-V	N	IH	57.1 (20.6)	0.006
*T* = 20	IA-N	IA-V	N	IA	54.9 (22.0)	0.01
	IH-V	IA-V	IH	V	34.6 (10.5)	0.001
	IH-N	IA-N	IH	N	73.8 (33.8)	0.029
	IH-N	IH-V	N	IH	94.1 (27.7)	0.001
*T* = 30	IA-N	IA-V	N	IA	57.9 (18.5)	0.002
	IH-V	IA-V	IH	V	44.2 (12.5)	<0.001
	IH-N	IA-N	IH	N	112.6 (35.1)	0.001
	IH-N	IH-V	N	IH	126.3 (32.4)	<0.001
*T* = 60	IA-N	IA-V	N	IA	45.8 (16.8)	0.006
	IH-V	IA-V	IH	V	48.2 (9.9)	<0.001
	IH-N	IA-N	IH	N	165.0 (38.1)	<0.001
	IH-N	IH-V	N	IH	162.6 (35.6)	<0.001
*T* = 90	IA-N	IA-V	N	IA	37.0 (16.9)	0.03
	IH-V	IA-V	IH	V	32.2 (8.3)	<0.001
	IH-N	IA-N	IH	N	132.0 (37.2)	<0.001
	IH-N	IH-V	N	IH	136.8 (34.2)	<0.001
*T* = 120	IA-N	IA-V	N	IA	27.1 (9.7)	0.005
	IH-V	IA-V	IH	V	24.3 (6.8)	<0.001
	IH-N	IA-N	IH	N	107.4 (31.7)	0.001
	IH-N	IH-V	N	IH	110.2 (31.0)	<0.001

IH, intermittent hypoxia; IA, intermittent air; N, nifedipine; V, vehicle.

† Effect assessed presents the primary variable (IH vs. N) against a background of IA or V

‡ mean Δ (SE) represents the difference in glucose values between group comparisons

* P value determined using multivariable mixed regression models.

**Table 4.** Effects of intermittent hypoxia and nifedipine on the insulin tolerance test

Time (min)	Group A vs. Group B		Effect Assessed^†^	Background	Mean Δ (SE)‡	*P* Value*

*T* = 0	IA-N	IA-V	N	IA	8.2 (9.8)	0.40
	IH-V	IA-V	IH	V	36.4 (13.4)	0.006
	IH-N	IA-N	IH	N	29.4 (14.6)	0.10
	IH-N	IH-V	N	IH	1.2 (20.02)	0.95
*T* = 10	IA-N	IA-V	N	IA	13.8 (8.5)	0.11
	IH-V	IA-V	IH	V	22.2 (13.0)	0.09
	IH-N	IA-N	IH	N	22.8 (16.6)	0.17
	IH-N	IH-V	N	IH	14.4 (19.3)	0.46
*T* = 20	IA-N	IA-V	N	IA	9.0 (10.2)	0.38
	IH-V	IA-V	IH	V	17.2 (8.5)	0.04
	IH-N	IA-N	IH	N	15.2 (8.9)	0.09
	IH-N	IH-V	N	IH	7.0 (6.9)	0.31
*T* = 30	IA-N	IA-V	N	IA	5.0 (10.1)	0.62
	IH-V	IA-V	IH	V	18.2 (8.3)	0.03
	IH-N	IA-N	IH	N	11.0 (8.5)	0.20
	IH-N	IH-V	N	IH	−2.2 (6.3)	0.73
*T* = 40	IA-N	IA-V	N	IA	4.2 (9.2)	0.65
	IH-V	IA-V	IH	V	14.0 (8.4)	0.09
	IH-N	IA-N	IH	N	12.2 (7.7)	0.11
	IH-N	IH-V	N	IH	2.4 (6.6)	0.72
*T* = 50	IA-N	IA-V	N	IA	14.5 (7.4)	0.36
	IH-V	IA-V	IH	V	17.4 (5.5)	0.70
	IH-N	IA-N	IH	N	6.6 (8.5)	0.001
	IH-N	IH-V	N	IH	3.8 (7.4)	0.12
*T* = 60	IA-N	IA-V	N	IA	−5.0 (5.5)	0.36
	IH-V	IA-V	IH	V	7.8 (7.2)	0.70
	IH-N	IA-N	IH	N	21.8 (7.6)	0.004
	IH-N	IH-V	N	IH	14.0 (9.0)	0.12
*T* = 90	IA-N	IA-V	N	IA	−8.0 (4.0)	0.06
	IH-V	IA-V	IH	V	12.2 (8.3)	0.14
	IH-N	IA-N	IH	N	51.2 (19.4)	0.008
	IH-N	IH-V	N	IH	31.0 (20.6)	0.13
*T* = 120	IA-N	IA-V	N	IA	−4.0 (3.6)	0.26
	IH-V	IA-V	IH	V	17.8 (11.9)	0.14
	IH-N	IA-N	IH	N	59.4 (27.9)	0.033
	IH-N	IH-V	N	IH	37.6 (30.1)	0.21

IH, intermittent hypoxia; IA, intermittent air; N, nifedipine; V, vehicle.

† Effect assessed is primary variable (IH vs. N) against a background of IA or V

‡ Mean Δ (SE) represents the difference in fasting values between groups

* P value determined using multivariable mixed regression models.

## Data Availability

Data will be made available upon reasonable request.

## NEW & NOTEWORTHY

The results of this study demonstrate the interaction between intermittent hypoxemia (IH) and nifedipine in a murine model. IH raises fasting glucose and insulin levels, with nifedipine exacerbating these disturbances. Glucose tolerance worsens when nifedipine is administered alone, and IH magnifies the impairment in glucose disposal. These findings raise the possibility of potential deleterious effects of L-type calcium channel blockers in patients with obstructive sleep apnea (OSA).
