# Supplementary material for: L-type calcium channel blockade worsens glucose tolerance and β-cell function in C57BL6/J mice exposed to intermittent hypoxia
Source: Am J Physiol Endocrinol Metab. Author manuscript; Available in PMC 2026 Apr 13. (PMC13075647; doi:10.1152/ajpendo.00423.2023)
Supplement: Supplemental Material [file NIHMS2158881-supplement-Supplemental_Material.pdf]

Supplementary Data Results and Tables

L-type Calcium Channel Blockade Worsens Glucose Tolerance and β-Cell Function in C57BL6/J Mice Exposed to Intermittent Hypoxia

Stanley M. Chen Cardenas[1], Tess A. Baker[2], Larissa A. Shimoda[3], Ernesto Bernal-Mizrachi[2] and Naresh M. Punjabi[4]

[1] Division of Endocrinology, Diabetes, and Metabolism. Johns Hopkins University School of Medicine. Baltimore, Maryland.
[2] Division of Endocrinology, Diabetes, and Metabolism, Diabetes Research Institute, University of Miami, Miller School of Medicine, Miami, Florida.
[3] Division of Pulmonary, Critical Care, and Sleep Medicine. Johns Hopkins University School of Medicine. Baltimore, Maryland.
[4] Division of Pulmonary, Critical Care, and Sleep Medicine, University of Miami, Miller School of Medicine, Miami Florida.

SUPPLEMENTAL RESULTS

Table S1 shows the weight trajectories in mice across the four groups over the five day exposure period. While intermittent hypoxia was associated with weight loss, no statistically significant differences were noted between groups indicating that the weight matching in the four groups was successful.

Table S2 describes the effects of intermittent hypoxia (IH) and intermittent air (IA) on fasting glucose and insulin levels. Mean group differences are shown for comparisons made in Figure 2.

The area under the glucose tolerance curves from Figure 3B and 3C are shown in **Table S3**.

Differences between groups in the glucose and insulin values between groups during the glucose stimulated insulin secretion from **Figure 4** are shown in **Table S4A**. Differences in the insulinogenic index are shown in **Figure 4C** and described **Table S4B**.

Differences in the insulin content, proinsulin content, proinsulin-to-insulin ratio, and islet-to-acinar ratio between the different groups of animals are shown in **Table S5**.

Table S1: Weight trajectories in mice as a function of condition† and drug‡ assignment.

Day	IA-V		IH-V		IA-N		IH-N		p-value*
1	31.73	(0.67)	32.62	(0.86)	32.48	(0.90)	33.00	(0.89)	0.75
2	31.38	(0.75)	31.09	(0.85)	31.34	(0.78)	31.53	(0.80)	0.98
3	30.63	(0.63)	30.79	(0.71)	30.98	(0.67)	31.11	(0.70)	0.96
4	30.28	(0.56)	30.64	(0.61)	30.58	(0.60)	30.98	(0.67)	0.88
5	30.18	(0.51)	29.90	(0.72)	30.49	(0.57)	30.17	(0.74)	0.93

†Condition: Intermittent air (IA) or intermittent hypoxia (IH). ‡Drug: Vehicle (V) or Nifedipine (N).
* p-valued determined by using a generalized linear model stratified by day.

Table S2: Effects of intermittent hypoxia and nifedipine on fasting glucose and insulin values

Group A	vs.	Group B	Effect Assessed†	Background	Mean Δ‡	(SE)	p-value*
**Fasting Glucose (mg/dl)**							
IA-N		IA-V	N	IA	5.5	(5.1)	0.28
IH-V		IA-V	IH	V	33.0	(5.8)	< 0.001
IH-N		IA-N	IH	N	36.1	(6.7)	< 0.001
IH-N		IH-V	N	IH	8.6	(7.3)	0.24
**Fasting Insulin (ng/ml)**							
IA-N		IA-V	N	IA	0.05	(0.09)	0.59
IH-V		IA-V	IH	V	0.53	(0.08)	< 0.001
IH-N		IA-N	IH	N	0.42	(0.09)	< 0.001
IH-N		IH-V	N	IH	-0.06	(0.07)	0.42

IH: Intermittent hypoxia; IA: Intermittent Air; N: Nifedipine; V: Vehicle
†Effect assessed presents the primary variable (IH vs. N) against a background of IA or V
‡Mean Δ (SE) represents the difference in fasting glucose and insulin values between groups.
*p-value determine using multivariable mixed regression models.

Table S3: Effects of intermittent hypoxia and nifedipine on Area Under the Curve (AUC) for Glucose and Insulin during the IPGTT and ITT

Group A	vs.	Group B	Effect Assessed†	Background	Mean Δ‡	(SE)	p-value*
AUC - IPGTT							
IA-N		IA-V	N	IA	0.49	(0.18)	0.006
IH-V		IA-V	IH	V	0.44	(0.10)	< 0.001
IH-N		IA-N	IH	N	1.42	(0.38)	< 0.001
IH-N		IH-V	N	IH	1.47	(0.35)	< 0.001
AUC - ITT							
IA-N		IA-V	N	IA	0.38	(0.48)	0.43
IH-V		IA-V	IH	V	1.04	(0.47)	0.03
IH-N		IA-N	IH	N	0.98	(0.52)	0.05
IH-N		IH-V	N	IH	0.32	(0.51)	0.52

IH: Intermittent hypoxia; IA: Intermittent Air; N: Nifedipine; V: Vehicle
†Effect assessed presents the primary variable (IH vs. N) against a background of IA or V
‡Mean Δ (SE) represents the difference in fasting glucose and insulin values between group comparisons
*p-value determine using multivariable mixed regression models.

Table S4A: Effects of intermittent hypoxia and nifedipine on glucose stimulated insulin secretion

Group A	vs.	Group B	Effect Assessed†	Background	Mean Δ‡	(SE)	p-value*
				**Glucose (mg/dl)**			
T=0 min							
IA-N		IA-V	N	IA	14.8	(10.5)	0.16
IH-V		IA-V	IH	V	37.2	(7.2)	<0.001
IH-N		IA-N	IH	N	31.9	(9.1)	<0.001
IH-N		IH-V	N	IH	9.4	(7.4)	0.20
T=15 min							
IA-N		IA-V	N	IA	52.1	(18.0)	0.004
IH-V		IA-V	IH	V	34.5	(13.0)	0.008
IH-N		IA-N	IH	N	59.0	(26.0)	0.023
IH-N		IH-V	N	IH	76.0	(23.6)	<0.001
T=30 min							
IA-N		IA-V	N	IA	67.2	(19.6)	0.001
IH-V		IA-V	IH	V	48.4	(14.6)	0.001
IH-N		IA-N	IH	N	108.4	(35.0)	0.002
IH-N		IH-V	N	IH	127.2	(33.1)	<0.001
				**Insulin (ng/ml)**			
T=0 min							
IA-N		IA-V	N	IA	0.02	(0.08)	0.76
IH-V		IA-V	IH	V	0.50	(0.05)	<0.001
IH-N		IA-N	IH	N	0.46	(0.07)	<0.001
IH-N		IH-V	N	IH	-0.01	(0.05)	0.79
T=15 min							
IA-N		IA-V	N	IA	-0.12	(0.09)	0.18
IH-V		IA-V	IH	V	0.52	(0.09)	< 0.001
IH-N		IA-N	IH	N	0.37	(0.09)	<0.001
IH-N		IH-V	N	IH	-0.27	(0.09)	0.002
T=30 min							
IA-N		IA-V	N	IA	0.09	(0.08)	0.23
IH-V		IA-V	IH	V	0.56	(0.07)	< 0.001
IH-N		IA-N	IH	N	0.18	(0.08)	0.02
IH-N		IH-V	N	IH	-0.29	(0.09)	0.001

IH: Intermittent hypoxia; IA: Intermittent Air; N: Nifedipine; V: Vehicle

†Effect assessed presents the primary variable (IH vs. N) against a background of IA or V

‡Mean Δ (SE) represents the difference in fasting glucose and insulin values between group comparisons

*p-value determine using multivariable mixed regression models

Table S4B: Effects of intermittent hypoxia and nifedipine on the insulinogenic index

Group A	vs.	Group B	Effect Assessed†	Background	Mean Δ‡	(SE)	p-value*
**Insulinogenic index**							
IA-N		IA-V	N	IA	-0.54	(0.59)	0.39
IH-V		IA-V	IH	V	0.72	(0.95)	0.47
IH-N		IA-N	IH	N	-1.80	(0.53)	0.003
IH-N		IH-V	N	IH	-3.06	(0.92)	0.003

IH: Intermittent hypoxia; IA: Intermittent Air; N: Nifedipine; V: Vehicle

†Effect assessed presents the primary variable (IH vs. N) against a background of IA or V

‡Mean Δ (SE) represents the difference in fasting glucose and insulin values between group comparisons

*p-value determine using multivariable mixed regression models

Table S5: Effects of intermittent hypoxia and nifedipine on insulin content, proinsulin content, proinsulin-to-insulin ratio, and islet-to-acinar ratio

Group A	vs.	Group B	Effect Assessed†	Background	Mean Δ‡	(SE)	p-value*
**Insulin Content ([ng/ml]/mg pancreas])**							
IA-N		IA-V	N	IA	-15.6	(9.2)	0.12
IH-V		IA-V	IH	V	-10.4	(7.1)	0.17
IH-N		IA-N	IH	N	8.4	(9.6)	0.40
IH-N		IH-V	N	IH	3.3	(7.6)	0.67
**Pro-Insulin Content ([ng/ml]/mg pancreas])**							
IA-N		IA-V	N	IA	-10.0	(45.2)	0.83
IH-V		IA-V	IH	V	-8.2	(25.9)	0.75
IH-N		IA-N	IH	N	12.1	(44.8)	0.79
IH-N		IH-V	N	IH	10.4	(25.2)	0.68
**Proinsulin to Insulin Ratio (nmol/mg pancreas)**							
IA-N		IA-V	N	IA	21.9	(12.9)	0.09
IH-V		IA-V	IH	V	5.7	(5.0)	0.25
IH-N		IA-N	IH	N	-13.4	(12.3)	0.28
IH-N		IH-V	N	IH	2.7	(3.1)	0.37
**Islet to Acinar Ratio**							
IA-N		IA-V	N	IA	-1.3	(2.6)	0.63
IH-V		IA-V	IH	V	-1.4	(2.8)	0.61
IH-N		IA-N	IH	N	-1.5	(0.9)	0.10
IH-N		IH-V	N	IH	-1.3	(1.3)	0.29

IH: Intermittent hypoxia; IA: Intermittent Air; N: Nifedipine; V: Vehicle

†Effect assessed presents the primary variable (IH vs. N) against a background of IA or V

‡Mean Δ (SE) represents the difference in fasting glucose and insulin values between group comparisons

*p-value determine using multivariable mixed regression models.
